# Sane Indications of the Uses of Medicinal Baths and Exercise

**Published:** 1915-10

**Authors:** Theodore Toepel

**Affiliations:** Atlanta, Ga.


### Journal-Record of Medicine

Successor to Atlanta Medical and Surgical Journal, Established 1855
and Southern Medical Record, Established 1870

**OWNED BY THE ATLANTA MEDICAL JOURNAL COMPANY**

PUBLISHED MONTHLY

*Official Organ Fulton County Medical Society, State Examing Board, Atlanta, Birmingham and Atlantic Railroad Surgeon's Association, Chattahoochee Valley Medical and Surgical Association, Etc.*

RICHARD R. DALY, M. D., Editor
BERNARD WOLFF, M. D., Supervising Editor
A. W. STIRLING, M. D., C. M., D. P. H.; J. S. HURT, B. Ph., M.D.
GEO. M. NILES, M. D., W. J. LOVE, M. D., (Ala.); Associate Editors
E. W. ALLEN, Business Manager

**COLLABORATORS**

W. F. WESTMORELAND, M. D., General Surgery
F. W. McRAE, M. D., Abdominal Surgery
ALLEN H. BUNCE, Medical Societies
E. B. BLOCK, M. D., Diseases of the Nervous System
MICHAEL HOKE, M. D., Orthopedic Surgery
CYRUS W. STRICKLER, M. D., Legal Medicine and Medical Legislation
E. C. DAVIS, A. B., M. D., Obstetrics
E. G. JONES, A. B., M. D., Gynecology
ALLEN H. BUNCE, M. D., Pathology and Bacteriology
R. T. DORSEY, Jr., B. S., M. D., Medicine
L. M. GAINES, A. B., M. D., Internal Medicine
GEO. C. MIZELL, M. D., Diseases of the Stomach and Intestines
L. B. CLARKE, M. D., Pediatrics
EDGAR PAULLIN, M. D., Opsonic Medicine
THEODORE TOEPEL, M. D., Mechano Therapy
R. R. DALY, M. D., Diseases of the Eye, Ear, Nose and Throat
BERNARD WOLFF, M. D., Diseases of the Skin
E. G. BALLENGER, M. D., Diseases of the Genito-Urinary Organs

VOL. LXII. ATLANTA, GA., OCTOBER, 1915. NO. 7

### SANE INDICATIONS OF THE USES OF MEDICINAL BATHS AND EXERCISE.

By THEODORE TOEPEL, M. D., Atlanta, Ga.

The use of cold water externally in the presence of fever is growing, thanks to Brand, Barnch, Winterwitz, and their disciples, but hydrotherapy has other and even more important applications; to secure the full benefit of its powers, apparatus more or less elaborate and special institutes are needed.

Recent developments point to a wider use of dry heat, of sunlight, and of various forms of artificial light and other radiations in the treatment of local and nutritional disorders.

In the judicious alternation of rest and exercise, applicable not alone to the amelioration of neurotic or debilitated states, but also to nearly all metabolic affections, one finds a method of treatment in harmony with the rhythmic alternations in nature. The now justly celebrated "Nauheim" or "Schott" methods for the treatment of cardiovascular disorders by means of thermal, saline, and carbonated baths, and gentle resistance exercises, afford a combination of the advantages of water, heat, mineral baths and mechanical measures, whose striking results are but illustrations of what can be accomplished for the relief even of patients affected with serious organic lesions, without the use of drugs.

Carbonated Baths. The action of carbonated baths upon the heart and circulation is of special importance. After immersion in the bath for from ten to fifteen minutes, an average decrease in the frequency of the pulse of from four to six beats a minute. This diminution persists for from fifteen to twenty minutes. The carbonated baths also increase the blood pressure. These baths also induce, through reflex influences, a more powerful action of the heart, a more vigorous pulse, with longer intervals; further, greater fullnes of the arterial system.

The carbonated baths are therefore indicated: In conditions of debility following acute diseases, after hemorrhage or other wasting discharge, in cases of neurasthenia and various disorders of the nervous system, in the presence of disease of the female genitalia, particularly derangements of menstruation. These baths have, however, been recommended especially in recent years in the treatment of the most varied kinds of cardiac insufficiency and diseases of the heart.

Brine Baths (Soolbaeder). The term brine baths is applied to those baths that are prepared from mineral waters containing sodium chlorid in such quantity that the specific gravity exceeds 1050.

The action of the brine bath depends, apart from the effect of the temperature, in the first place, upon the chemical irritation that the concentrated solution of sodium chlorid exerts upon the skin and its nerves.

With regard to the influence of the brine bath upon the metabolism it has been found that three per cent. salt baths at a temperature of 95° F. for a period of thirty minutes, caused a distinct diuretic effect, an inconsiderable increase in the chlorids, a slight diminution in the elimination of nitrogen. Brine baths are indicated in cases of scrofulosis and rachitis, with their tendency to chronic inflammation, in cases of rheumatoid arthritis, exudates of various kinds, etc.

Thermal Brine Baths—that is those that have a high temperature—possess in this thermal quality and in the presence of carbon dioxid material advantage over the cooler brine baths. The thermal brine baths are especially indicated when it is desired to improve the nervous forces, as well as the general nutrition of the body; and they are accordingly recommended especially in cases of paralysis, neuroses, tabes dorsalis, and spinal irritations.

Electric Light Baths. The effects of the electric light upon the skin are the same as those induced by the sun—profuse perspiration, pigmentation, and erythema. Patients, however, begin to perspire more quickly than when exposed to an ordinary sun-bath.

The therapeutic effects of the electric light bath may be summarized briefly as follows:

1. It is one of the most effective of all means of producing general and local revulsive effects, by dilating the cutaneous vessels.

2. The electric light bath certainly has no equal among therapeutic means as a sudorific measure; it induces perspiration more quickly and more vigorously than any other agent, and with the least amount of inconvenience and discomfort to the patient.

3. It is a most effective means of promoting the absorption of exudates. For this purpose both general and local applications are valuable.

4. For tonic effects no other means excels short applications of electric light. A sensation of well-being is most pronounced, and when followed by a proper hydriatic application, the stimulation to nutrition is of the highest possible degree.

Indications. Diabetes and obesity are benefited by the electric light bath through its potent influence upon carbon dioxid formation by the stimulation of the oxidation processes within the body. Fat diabetics are especially likely to be benefited by the electric light bath.

Obesity is in a high degree amenable to the powerful influence of the electric light.

Sciatica, intercostal neuralgia, vague neuralgic pains, and myalgia yield readily, in the majority of cases, to the use of the electric light bath, especially when combined with carefully administered tonic hydriatic measures, massage, a proper dietary, and an outdoor life.

On chlorosis and anemia the most excellent results are obtained by the systematic employment of the electric light bath. There is no more efficient means of dilating the cutaneous vessels, to whose contraction is due the pallor characteristic of these disorders, thus relieving the visceral congestion necessarily present.

In nephritis the electric light bath, by diverting half to two-thirds of all the blood in the body into the skin, affords prompt relief of the congested and inflamed parts.

A condensed abstract from an address by Dr. Curtis F. Burman at the Johns Hopkins Medical School's twenty-fifth anniversary celebration of "The Status of Radium Therapeutics."

Radium is a metallic element belonging to the strontium barium group. It readily forms salts with mineral acids and is the leading member of the peculiar radio-active group of elements which are characterized by atomic instability. Radium itself is formed by atomic reduction from uranium. It loses a portion of its atom to become a gas called emanation, and this in turn is the mother of a series of solid elements.

The essential characteristic of the radio-active substances is the giving off of invisible rays. These rays must not be confused with the emanation, which is an element just as radium itself is. The rays have been divided according to their physical characteristics into three kinds: the alpha, the beta and the gamma

The internal use of radium or of radium emanation, which being a gas can be readily taken up through the lungs, has been principally investigated in Germany. There seems no question but that certain forms of gout, rheumatism, chronic arthritis neuralgia can be greatly helped by its use. It has a pronounced effect upon the hemaporictic tissues, in small doses stimulating, and in large doses destroying them. Unquestionable improvements have followed its use in cases of pernicious anemia, leukemia and arythro-polycythemia; likewise in certain patients suffering with high blood pressure, a return to the normal with complete disappearance of subjective symptoms being frequently obtainable.

Physiology of Exercise. The contraction of the muscle substances is accomplished by a mechanism as yet beyond ultimate analysis.

Effect upon the Muscle-cell. Some of the highly complex cell-constituents are broken down and extruded. The cell at once proceeds to absorb from the surrounding plasma additional food materials, particularly carbohydrates and oxygen. Thus, muscular activity results in the constant change of some of the essential elements of the muscle-cell itself, which is kept in a state of increased efficiency proportionate to its use.

General Effects of Muscular Exercise. The absorption by the muscle-cells of oxygen and carbohydrates from the blood plasma has immediate and ultimate effects upon the body as a whole. When muscular energy is expended, the blood is altered in constitution. Such blood flowing through the respiratory center in considerable quantities causes increased respiratory movements. All the muscles or respiration are thus brought into immediate and often vigorous action, even the accessory muscles being called on promptly when there is particular need of rapidly augmenting the oxygen supply. The blood circulating through the lungs makes up its oxygen tension, the added activity of respiration changes the air in the lungs, with the result that during exercise the percentage of oxygen in the lungs is greater than usual. The contraction of the muscular fibers squeezes the lymph-vessels and the smaller veins so that blood and lymph are pressed onward toward the heart. This, together with stimulation of the centers governing circulation in the medulla, causes increased cardiac activity. The quickening and deepening of respiration also are effective in the aspiration of the thorax. By these various means the circulation of the blood is at once markedly stimulated, and the arteries supplying the muscles exercised are immediately somewhat dilated. We thus have a completely adjusted mechanism for augmenting food-supply and eliminating waste. The muscle-cell eliminates the products of combustion in the form of carbon dioxid and the urea or some of its antecedents. These are carried away by the circulation, which is now accelerated, the carbon dioxid being eleminated by the lungs, the urea by the kidneys. The greater consumption by the cell of carbohydrates and proteids affects the organism in ways that are somewhat analogous to the enlarged need of oxygen, but instead of causing oxygen hunger there is produced hunger for food. Through the influence again of the vasomotor system, the whole digestive tract comes eventualy into a state of heightened activity, in part owing to the increased blood supply, but chiefly to direct neural stimulation. The consciousness of hunger is more marked, and gradually the normal individual will be led to eat more food as certainly and as definitely as he is prompted to breathe more air. A third group of activities is due to the heat evolved by the rapid combustion in the muscles. This is kept down by the dilatation of the superficial arteries and capillaries which increases surface radiation. At the same time the sweat glands are influenced through the sympathetic nervous system to operate with more or less vigor, throwing sweat upon the skin. The evaporation of this sweat cools the body, and is a potent factor in preventing the rise of the body temperature. Thus, through muscular exercise, the function of respiration, circulation, nutrition, and excretion are all profoundly affected, and the heat-controlling mechanism of the skin and sweat-glands are stimulated to greater action.

1626 Candler Building, Atlanta, Ga.

